# Intestinal Schistosomiasis: A Rare Cause of Abdominal Pain and Weight loss

**DOI:** 10.7759/cureus.2086

**Published:** 2018-01-18

**Authors:** Asim Shuja, Jian Guan, Ciel Harris, Ahmad Alkhasawneh, Miguel Malespin, Silvio De Melo

**Affiliations:** 1 Division of Gastroenterology, University of Florida College of Medicine-Jacksonville; 2 Internal Medicine, Florida Hospital-Orlando; 3 Internal Medicine, University of Florida College of Medicine-Jacksonville; 4 Pathology, University of Florida College of Medicine-Jacksonville

**Keywords:** intestine, schistosomiasis

## Abstract

Abdominal pain is one of the most common reasons for outpatient visits. Although intestinal schistosomiasis is extremely rare in US, it should be considered in the differential diagnosis for those patients with risk factors such as international traveling history. This case report illustrates a unique case of intestinal schistosomiasis, which presented with an eight-week history of nonspecific abdominal pain and weight loss. Her colonoscopy revealed a 10 mm polyp in the colon. Endoscopic mucosal resection confirmed the diagnosis of schistosomiasis. Treatment with Praziquantel resulted in significant improvement of her symptoms.

## Introduction

Chronic or recurrent abdominal pain has been one of the most challenging dilemmas in the outpatient and inpatient arenas. The prevalence of chronic or recurrent abdominal pain in adults is estimated to be almost 50% [1]. The workup for chronic/recurrent abdominal pain can be a daunting task due to the innumerable etiologies. A thorough history and physical examination is often helpful to identify alarming features and guide further investigation.

As a worldwide parasitic disease, schistosomiasis currently poses risk to over 230 million patients in more than 70 countries, resulting in 300,000 deaths annually in Africa alone [2-4]. Schistosomiasis is extremely rare in the United States and almost all patients with schistosomiasis reported in the U.S. are related to international travel to endemic countries. The mechanism by which schistosomiasis causes symptoms is reported to be due to a granulomatous reaction from the trapped eggs interacting with host immunity. The symptoms of intestinal schistosomiasis, includes diarrhea, abdominal pain, dyspepsia, and malnutrition, and they are non-specific. Therefore, definitive diagnosis of intestinal schistosomiasis requires egg identification from fecal and/or urine samples or biopsy via colonoscopy. Early diagnosis and treatment of schistosomiasis is important in preventing serious complications, such as anemia, chronic ulceration, bowel stricture, and obstruction.

## Case presentation

**Figure 1:**
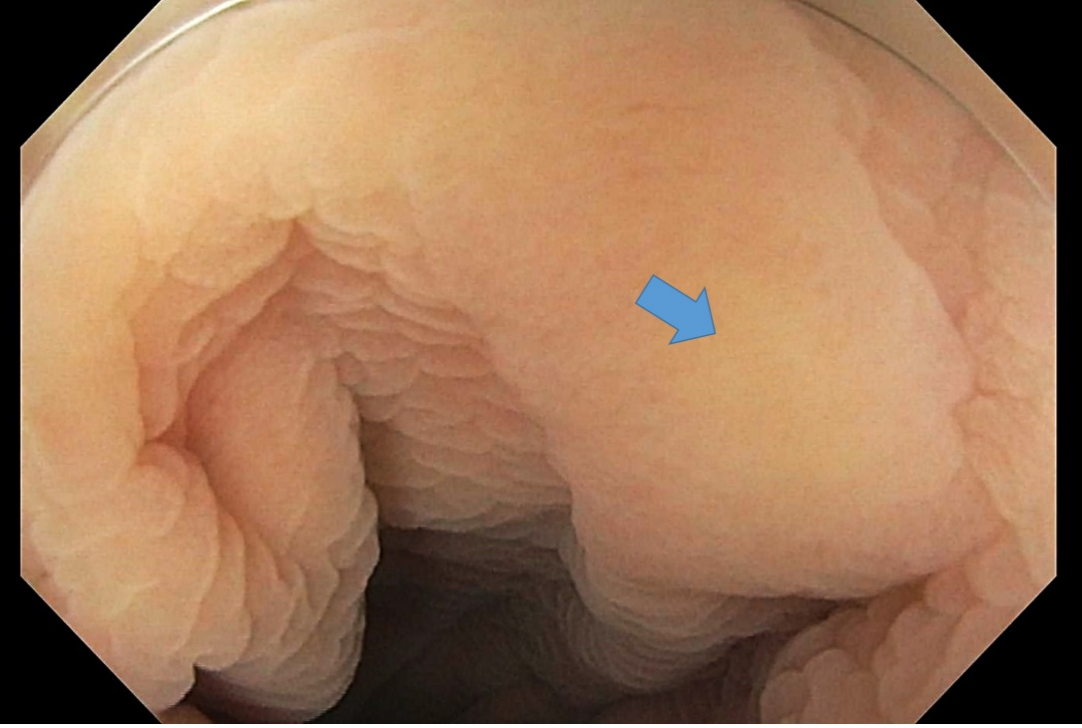
Gross image of a large flat polyp in descending colon

**Figure 2:**
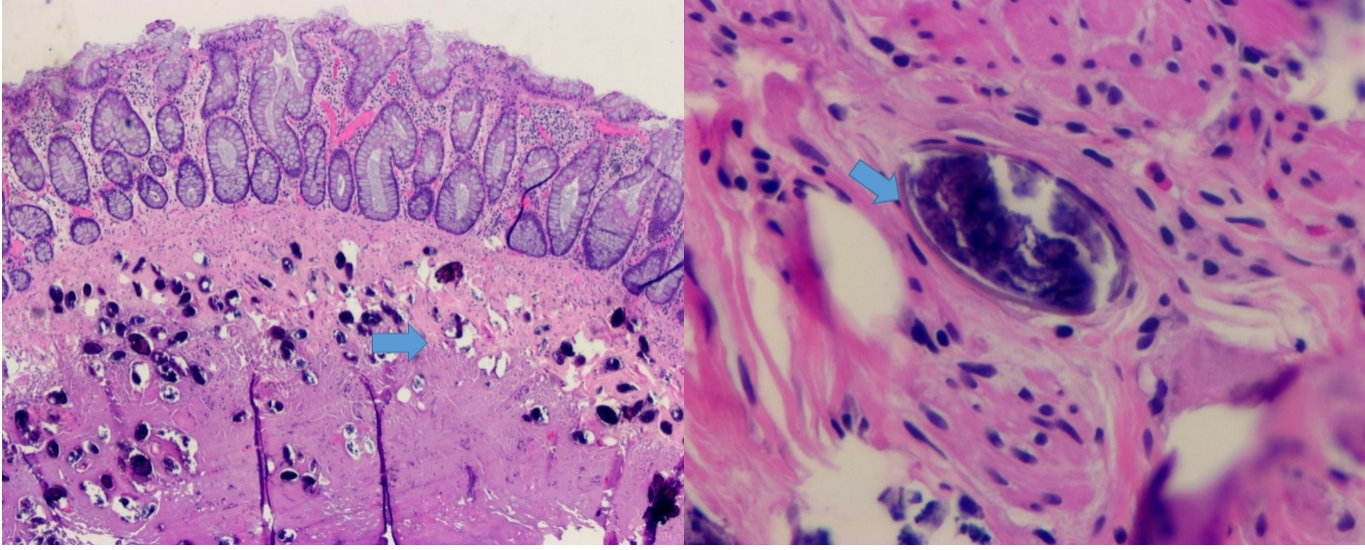
H&E staining of a resected polyp showing the numerous eggs deposition in the submucosal space

We report a 63-year-old Asian female with no significant past medical history, who presented to our clinic reporting an eight-week history of abdominal pain, anorexia, and weight loss. The abdominal pain was mild to moderate, crampy in nature and generalized. It was aggravated with food intake and partially relieved with proton pump inhibitors and probiotics. It was associated with nausea, bloating and an unintentional weight loss of five kilograms in six months. She denied any fever, skin rash, chronic diarrhea or rectal bleeding. She reported traveling back and forth from Hawaii to the Philippines many times before she moved to Florida one year prior. Her physical examination was unremarkable. Her complete blood count and metabolic profile were unremarkable except for a borderline normocytic anemia (hemoglobin at 11.7g/dL). Urine analysis and routine ova and parasites exams in both urine and stool were negative. Right upper quadrant ultrasound was unrevealing. Esophagogastroduodenoscopy (EGD) was unremarkable, as shown in Figure 1, while colonoscopy showed a 10 milimeter (mm) flat polyp in the descending colon which underwent endoscopic mucosal resection (EMR). Pathology showed hyperplastic colon mucosa with numerous parasite eggs deposition in the submucosa, consistent with Schistosoma species (Figure 2). The patient was then treated with Praziquantel (1800 mg in divided doses for one day) and her symptoms, including abdominal pain and bloating, were reported to improve. She also started to gain weight as noted in the two-week follow up visit.

​​​​​​​

## Discussion

Schistosomiasis is a parasitic disease which is caused by trematode flukes Schistosoma includingS. haematobium, S. mansoni and S. japonicum [5]. Schistosomiasis is a leading global health issue affecting more than 200 million people worldwide, particularly with the epidemic spreading in topical areas. The lifecycle of schistosomiasis starts with eggs in the urine or faeces released to freshwater environment from an infected host. After hatch, miracidia typically infect the intermediate host snail, in which infectious cercariae are maturated, amplified, and eventually released into freshwater. Acute schistosomiasis is typically a self-limited process seen in travelers caused by a cercariae induced hypersensitivity reaction [6]. It typically presents as fever, maculopapular skin rash, abdominal pain, and generalized myalgia. Chronic schistosomiasis is featured with egg induced granulomatous inflammation, resulting in fibrosis, obstruction and even possible precancerous pathology changes [7]. Among many organs involved in chronic schistosomiasis, intestinal, hepatic, and urinary are the most well documented. 

Polyp formation is one of the most frequently intestinal lesions seen in chronic intestinal schistosomiasis [8]. The underlying mechanism starts with egg deposition in the loose superficial layers of submucosa and subsequently progresses into cell mediated inflammatory response with granuloma formation to form polyps [7]. It frequently presents as abdominal pain, diarrhea, tenesmus and weight loss, all of which are not specific to intestinal schistosomiasis. The diagnosis of intestinal schistosomiasis typically requires egg identification from stool samples or endoscopy biopsy. Serology to detect anti-schistosomal antibodies in serum or urine can be helpful to rule out infection in the epidemic area but its sensitivity and specificity can sometimes be challenging in the non-epidemic area [7]. Polymerase chain reaction (PCR) to detect the free circulating DNA from schistosomiasis is a quick and more sensitive method but its clinical usefulness remains to be determined and is not readily available in all U.S. hospitals [9]. Treatment for schistosomiasis is relatively safe and effective, especially in the developed countries where the resistance to Praziquantel (PZQ) has not been reported yet [7].

Despite the fact that schistosomiasis is second only to malaria in terms of its socioeconomic impact on global health, it is often neglected in U.S. Intestinal schistosomiasis should be considered in the differential diagnosis for chronic abdominal pain and weight loss, especially in patients with a history of travel to endemic areas. In our case, the identification of colonic polyp consisting of schistosoma eggs, absence of other intra-abdominal pathology, and the improvement of her symptoms with anti-schistosomiasis treatment strongly imply the diagnosis of intestinal schistosomiasis. We believe that our case is probably the only patient with intestinal schistosomiasis presenting with abdominal pain and polyps that has ever been reported in U.S. [10]. As shown in our patient and literature, clinical symptoms and laboratory tests are typically not specific enough to aid the diagnosis of intestinal schistosomiasis, therefore, careful history acquisition and egg identification are crucial to establish early diagnosis and prevent complications.

## Conclusions

Despite the fact that intestinal schistosomiasis is extremely rarely reported in the U.S., it should be considered in the differential diagnosis for chronic unspecific abdominal pain given increasing international travel resulting from globalization. We present a unique case of intestinal schistosomiasis in an American with eight weeks’ history of nonspecific abdominal pain and weight loss. Egg identification via endoscopic mucosal resection and quick response to Praziquantel confirm the diagnosis of intestinal schistosomiasis. Eliciting a detailed social and traveling history is crucial for establishing a diagnosis and early treatment. 
